# Transcriptional Profiling of the Circulating Immune Response to Lassa Virus in an Aerosol Model of Exposure

**DOI:** 10.1371/journal.pntd.0002171

**Published:** 2013-04-25

**Authors:** Shikha Malhotra, Judy Y. Yen, Anna N. Honko, Sara Garamszegi, Ignacio S. Caballero, Joshua C. Johnson, Eric M. Mucker, John C. Trefry, Lisa E. Hensley, John H. Connor

**Affiliations:** 1 Department of Microbiology, Boston University School of Medicine, Boston, Massachusetts, United States of America; 2 U.S. Army Medical Research Institute of Infectious Diseases, Fort Detrick, Maryland, United States of America; 3 Graduate Program in Bioinformatics, Boston University, Boston, Massachusetts, United States of America; 4 Integrated Research Facility at Fort Detrick, Division of Clinical Research, National Institute of Allergy and Infectious Diseases, National Institutes of Health, Frederick, Maryland, United States of America; University of Texas Medical Branch, United States of America

## Abstract

Lassa virus (LASV) is a significant human pathogen that is endemic to several countries in West Africa. Infection with LASV leads to the development of hemorrhagic fever in a significant number of cases, and it is estimated that thousands die each year from the disease. Little is known about the complex immune mechanisms governing the response to LASV or the genetic determinants of susceptibility and resistance to infection. In the study presented here, we have used a whole-genome, microarray-based approach to determine the temporal host response in the peripheral blood mononuclear cells (PBMCs) of non-human primates (NHP) following aerosol exposure to LASV. Sequential sampling over the entire disease course showed that there are strong transcriptional changes of the immune response to LASV exposure, including the early induction of interferon-responsive genes and Toll-like receptor signaling pathways. However, this increase in early innate responses was coupled with a lack of pro-inflammatory cytokine response in LASV exposed NHPs. There was a distinct lack of cytokines such as IL1β and IL23α, while immunosuppressive cytokines such as IL27 and IL6 were upregulated. Comparison of IRF/STAT1-stimulated gene expression with the viral load in LASV exposed NHPs suggests that mRNA expression significantly precedes viremia, and thus might be used for early diagnostics of the disease. Our results provide a transcriptomic survey of the circulating immune response to hemorrhagic LASV exposure and provide a foundation for biomarker identification to allow clinical diagnosis of LASV infection through analysis of the host response.

## Author Summary

Lassa virus (LASV), a member of the *Arenaviridae* family, is a viral hemorrhagic fever causing virus endemic to several countries in West Africa with a history of sporadic importation into the United States. It has been characterized as a Category A agent, and despite the significant public health issues posed by LASV and the potential biodefense risks, little is known about the immune response to the virus. In the study presented here, we have taken an unbiased genomics approach to map the temporal host response in the peripheral blood mononuclear cells (PBMCs) of non-human primates (NHP) exposed to LASV. Gene expression patterns over the entire disease course showed that there are strong transcriptional changes of the immune response to LASV exposure, including the upregulation of Toll-like receptor signaling pathways and innate antiviral transcription factors. However, there was a lack of pro-inflammatory cytokine response in LASV exposed NHPs similar to what is seen in human disease. Our data suggests that LASV induces negative regulation of immunological events, leading to an inefficient adaptive immune response as observed in LASV-infected human patients. Our results provide a picture of the host's circulating immune response to hemorrhagic LASV exposure and demonstrate that gene expression patterns correlate with specific stages of disease progression.

## Introduction

Lassa virus (LASV) is a segmented negative-strand RNA virus and a member of the Arenavirus genus. LASV is a human pathogen that is endemic to several countries in West Africa. It is estimated to infect more than 300,000 people each year, killing over 3,000 with fatality rate for Lassa fever (LF) being approximately 15% in hospitalized patients [1]. In several outbreaks 50% case fatality have been reported [2]. LF was initially described as Lassa hepatitis and liver pathology is a significant histological finding in LF patients [3] and in animal models of LF infection [4]. The natural host of LASV is a highly commensal rodent, *Mastomys natalensis* [5], [6], and infection with LASV is thought to occur by direct contact with the host or via aerosol. In part because of its ability to be transmitted through aerosol means [7], as well as potential for high lethality, LASV has been characterized as a Category A bioweapon agent [8].

There are currently no FDA-approved vaccines or antiviral drugs to treat LASV infection. Ribavirin treatment has been suggested to reduce morbidity in infected patients [9] when initiated within few days of disease onset [10]. Ribavirin is often poorly tolerated and has been associated with a number of severe adverse events. Given the number of side effects associated with this drug, the potential for severe adverse events, and the limited efficacy, there is a strong need for more effective and safer drugs as well as vaccines. In order to develop and test candidate countermeasures, it is critical that there be well-characterized animal models that accurately reflect the human disease.

Currently there are several animal models for LASV infection. These include humanized mice expressing human HLA-A2.1 instead of murine MHC Class I gene [11], IFN receptor knock-out [12] mice that can be infected with LASV, and Strain 13 guinea pigs. None of these models completely reproduce the human disease [13]. As a result, more emphasis has been placed on several NHP species that are susceptible to LF [14]–[21]. Both African green monkeys and rhesus macaques show a lethal response to low challenge doses but only a partial response to high challenge doses [14], [22]. Cynomolgus macaques have been shown to be uniformly susceptible to lethal LASV infection at low and high challenge doses [4], [16], [17], [23].

The development and characterization of these animal models has facilitated the examination of host responses to LASV exposure. In the work presented here, we have used a whole genome microarray-based approach to determine the temporal host response to exposure in PBMCs from cynomolgus macaques following aerosol LASV exposure. Sequential sampling throughout the disease course provided us the opportunity to characterize the circulating immune response to LASV during different stages of infection. Furthermore, we were able to refine this characterization through analysis of immune cell subsets to define the responses of specific effector cells. These analyses showed that there are both rapid and delayed transcription events following LASV exposure, including the upregulation of Toll-like receptor signaling pathways and innate antiviral transcription factors. Our data show that the immune response to LASV involves the expression of a large number of immunosuppressive events in exposed NHPs leading to an inefficient adaptive immune response observed in LASV infections.

## Materials and Methods

### Animals

#### Aerosol model efficacy study

A confirmation of lethality study with four NHPs was done prior to the sequential sampling study. In this confirmation of lethality study, animals were exposed to a target dose of 1000 PFU of LASV Josiah in a head-only chamber in a class III biological safety cabinet maintained under negative pressure within a biosafety level 4 (BSL-4) laboratory. Aerosols were created by a 3-jet Collison nebulizer (BGI, Inc., Waltham, MA) and controlled by the automated bio-aerosol exposure system. Animal research was conducted at the United States Army Medical Research Institute of Infectious Diseases (USAMRIID). All experiments involving the use of LASV in animals were performed in USAMRIID's BSL-4 laboratory.

#### Sequential sampling study

Samples used for microarray analysis were taken from a larger sequential sampling study performed to characterize the disease progression of LASV after aerosol exposure (A. Honko et al, manuscript in preparation). For this study, fourteen adult male and female cynomolgus macaques (*Macaca fascicularis*), ranging in weight from 5.5 to 8.7 kg were obtained from licensed and approved vendors. This manuscript describes microarray analysis of eleven out of the fourteen NHPs in the study. The reason for this is provided under the section RNA Processing and DNA Microarrays in materials and methods. During the acclimation to the biosafety level 4 (BSL4) containment suite, a pre-challenge blood sample (at day −8) was collected to provide a baseline for further analyses. On day 0, NHPs were anesthetized and minute volumes calculated by performing whole-body plethysmography (Buxco Research Systems, Wilmington, NC) just prior to the exposure. NHPs were exposed individually at a target dose of 1,000 Plaque Forming Unit (PFU) of LASV Josiah in a head-only chamber in a class III biological safety cabinet maintained under negative pressure within a biosafety level 4 (BSL-4) laboratory. Aerosols were created by a 3-jet Collison nebulizer (BGI, Inc., Waltham, MA) and controlled by the automated bio-aerosol exposure system. The virus preparation used to infect NHPs was free of contamination of endotoxin and mycoplasma. After challenge, blood samples were collected at various days post-exposure (dpe), based on approved collection allowances, as well as at euthanasia. Groups of two to three NHP were euthanized at 3, 6, 8, 10, and 12 dpe and PBMCs prepared from blood collected. Serum chemistry analysis was performed according to the manufacturer's guidelines using a Piccolo Xpress Chemistry Analyzer (Abaxis, Union City, CA) on a Piccolo General Chemistry 13 reagent disc.

Both studies were carried out in accordance with standards of IACUC approved protocol in compliance with the regulations outlined in the USDA Animal Welfare Act (PHS Policy), and other Federal statutes and regulations relating to animals and experiments involving animals. The facility where this research was conducted is accredited by the Association for Assessment and Accreditation of Laboratory Animal Care, International and all animal work done adhere to the conditions specified in the *Guide for the Care and Use of Laboratory Animals* (National Research Council, 2011). These experiments and procedures were approved by the USAMRIID Institutional Animal Care and Use Committee (IACUC). Animals were given enrichment regularly as recommended by the *Guide for the Care and Use of Laboratory Animals*. The animals were fed and checked at least daily according to the protocol. All efforts were made to minimize painful procedures; the attending veterinarian was consulted regarding painful procedures, and animals were anesthetized prior to phlebotomy. Following the development of clinical signs, animals were checked multiple times daily. When clinical observations and scores of animals reached defined levels based on the approved IACUC protocol, animals were euthanized under anesthesia to minimize pain and distress.

### RNA processing and DNA microarrays

**Figure 1:**
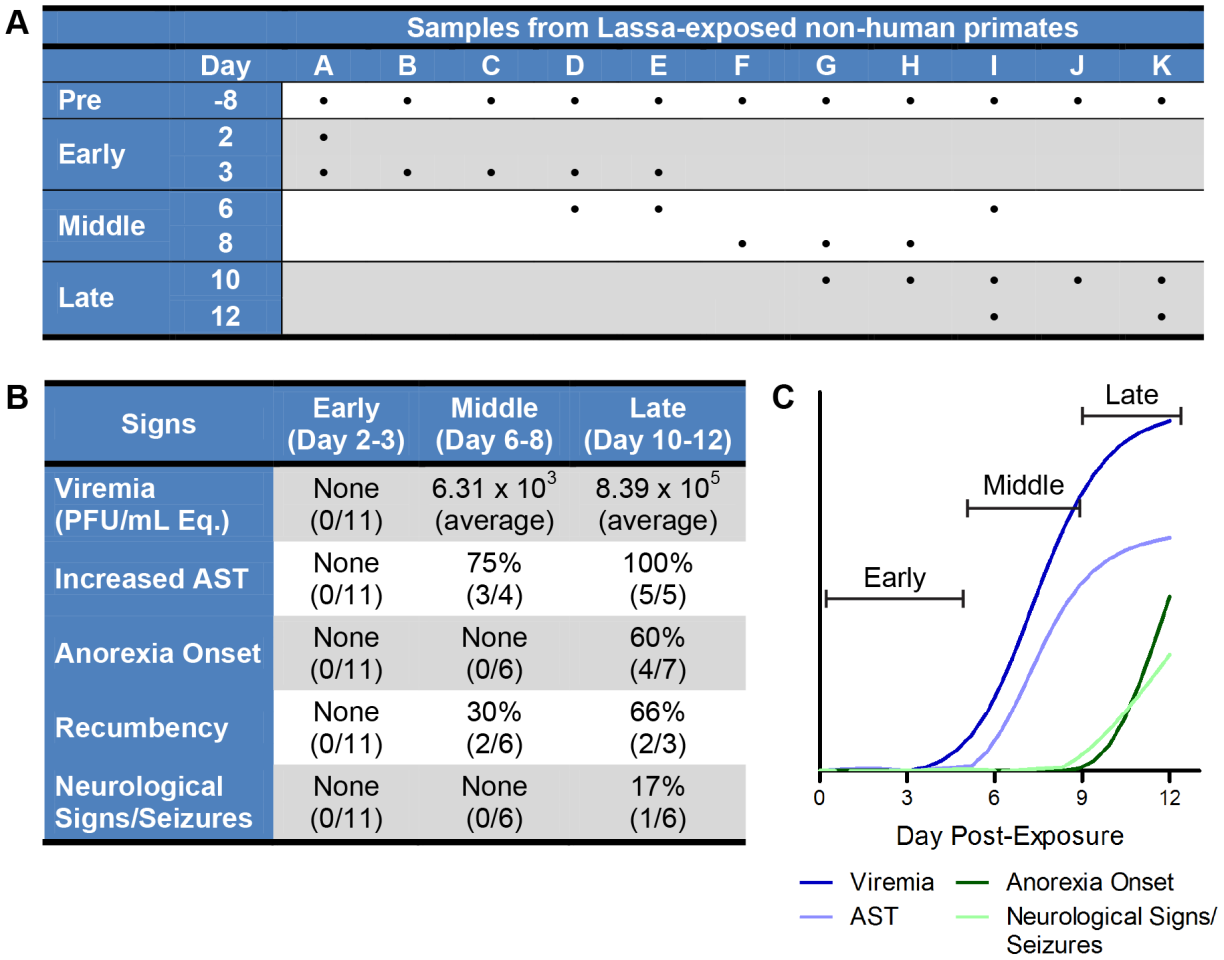
Confirmation of LASV aerosol model. (A) Overview of the sequential sampling study samples. The table is organized by animal (column, represented with letter A–K) and day that the sample was collected (row). Samples from LASV-exposed NHP were sorted temporally by the day the sample was collected post-exposure, and further divided into three general categories (early, middle, and late induction) to ease interpretation. Pre-exposure samples were collected prior to LASV exposure and used to normalize data obtained from subsequent samples from the same animal. (B) Table describes the time of onset (early, middle, and late) of a given symptom (viremia, increased AST, anorexia onset, recumbency, and neurological signs/seizures) post-LASV exposure. Data is presented as the number and percentage of animals in which a clinical finding was detected, followed by the total number of animals that were examined at a given stage. (C) A conceptual diagram to explain the division of early, middle, and late stages of LASV in our sequential sampling NHP model. Early disease stage (0–5 dpe) is asymptomatic; middle disease stage (6–9 dpe) is early symptomatic with increase in viremia (dark blue line) and AST levels (light blue line); late disease stage (10–12 dpe) is depicting increasing signs of disease with increase in the onset of anorexia (dark green line) and neurological signs/seizures (light green line) in NHPs.

Peripheral blood mononuclear cells (PBMCs) were isolated from blood pre-diluted with saline using ACCUSPIN System-Histopaque-1077 tubes as per manufacturer's recommendations, and subsequently lysed in TRI Reagent LS (Sigma-Aldrich) at USAMRIID. PBMCs were processed for microarray analysis as described earlier [24]. Briefly, total RNA was extracted from the TRI Reagent LS samples, then amplified using the Low-Input Quick Amp Labeling kit (Agilent) and hybridized to Whole Human Genome Oligo Microarrays (Agilent) in a 2-color comparative format along with a reference pool of messenger RNA (mRNA). Images were scanned using the Agilent High-Resolution Microarray Scanner and raw microarray images were processed using Agilent's Feature Extraction software. The quality of the microarray hybridization for pre-exposure samples obtained from three out of fourteen NHPs in the study was lower than needed for background corrections and normalization. Thus, DNA Microarray data from these three NHPs was not used for data analysis. In summary, out of the 46 samples (from fourteen NHPs) hybridized to microarrays, a subset of 30 (from eleven NHPs) were used in the subsequent analyses as shown in Figure 1A. The resulting microarray dataset has been submitted to the Gene Expression Omnibus (GEO) database, under series record GSE41752.

### Cell separation

Subsets of immune cells (CD4+, CD8+, CD14+, and CD20+) were separated from PBMCs by sequential positive selection using nonhuman primate microbeads (Miltenyi Biotec, Auburn, CA) as per the manufacturer's recommendations. Although cell separation procedures can cause activation that may affect transcriptional changes, in our study, a number of steps were taken to mitigate these effects. Cells were kept cold, and pre-chilled buffers were used to reduce nonspecific antibody binding, cell surface capping and activation. Additionally, all the pre-exposure (day −8) as well as post-exposure samples were separated using same separation procedure, and all changes in transcription of the pre-exposure samples were subtracted from the post-exposure samples. Therefore, we believe there was little contribution of the separation procedure on the observed transcription. Following incubation with FcR blocking reagent, CD20+ cells were isolated using MS columns and the flow-through fraction was then utilized for the CD14+ isolation. The CD14 flow-through fraction was used for the CD4+ isolation, and the CD4 flow-through fraction for the CD8+ isolation. To increase the purity of the CD20+ and CD14+ magnetically labeled fractions, these were passed over two prepared columns. Aliquots from positive fractions were retained for determination of cell numbers as well as assessment of purity using flow cytometry; the remainder was lysed in TRI Reagent LS.

### DNA microarray data processing

Data were first background-corrected to remove noise from background intensity levels, and afterwards were normalized within the arrays using the Limma package in R [25]. After normalization, the reference and experimental samples were compared to generate log2 fold-change values that represent a change in mRNA expression (either positive or negative). At this step, the internal array control probes were removed. Each array was then further normalized using the pre-exposure control array for that animal to remove monkey-specific expression changes from baseline. A comparison of gene expression was done for day −8 and day 0 samples and there seems to be no difference in the gene expression (data not shown). The resulting dataset was filtered for differential expression and annotated with gene names. The dataset was hierarchically clustered using the Cluster 3.0 [26] and visualized using Java Treeview [27]. Functional annotations of gene clusters were assigned using the Database for Annotation, Visualization and Integrated Discovery (DAVID) (http://david.abcc.ncifcrf.gov/) [28]. The *p*-values reported are the value reported by DAVID and are based on the EASE score. The EASE score is an alternative name of Fisher Exact Statistics used in the DAVID system, referring to a one-tail Fisher Exact Probability Value for gene-enrichment analysis.

### Cytokine detection by multiplex analysis

Cytokines were assayed in the plasma of LASV exposed NHPs using a NHP magnetic 23-plex multiplex assay (Millipore EMD) in accordance with manufacturer's instructions. Briefly, samples from both pre- and post-exposure time points were assayed in triplicate and washed using a Bio-Rad Bio-Plex Pro II Wash Station equipped with a magnetic manifold. Data were acquired using a Bio-Rad Bio-Plex 3D system and analyzed using Bio-Plex Manager 6.0 software and a 5-parameter logarithmic fit. Cytokine levels in assayed samples were derived from the standards run for each assay plate and presented as plasma cytokines in pg/mL. Cytokines/chemokines assayed included granulocyte-macrophage colony-stimulating factor (GM-CSF), interferon gamma (IFNγ), Interleukin (IL)-1 beta (IL1β), IL1 receptor antagonist (IL1RA), IL2, IL4, IL5, IL6, IL8, IL10, IL12/23(p40), IL13, IL15, IL17, IL18, monocyte chemoattractant protein-1 (MCP1), macrophage inflammatory protein (MIP)-1 alpha (MIP1α), MIP-1 beta (MIP1β), transforming growth factor-alpha (TGFα), vascular endothelial growth factor (VEGF), sCD40L, and tumor necrosis factor-alpha (TNFα).

### Detection of viral RNA by RT-PCR

RNA was isolated from serum of LASV exposed NHPs prepared with TRI Reagent LS (Sigma-Aldrich). The aqueous phase was extracted using Phase Lock heavy gel tubes (5 Prime) and 1-Bromo-3-chloropropane (BCP, Sigma-Aldrich) and mixed with 70% ethanol. Following 5 min incubation at room temperature, it was added to an RNeasy column and extracted according to the manufacturer's recommendations (QIAGEN). RNA was eluted through two consecutive additions of 50 µL of nuclease-free water and stored at −80°C until analysis. One-step quantitative real-time RT-PCR reactions were performed on a LightCycler 480 (Roche, Indianapolis, IN, USA) in 20 uL volumes with 5 uL of purified RNA and the Superscript II One-Step RT-PCR System (Life Technologies). Primers and probe were specific for the LASV GP gene [Forward, 900 nM: TgCTAgTACAgACAgTgCAATgAg; Reverse, 900 nM: TAgTgACATTCTTCCAggAAgTgC (Oligos Etc., Wilsonville, OR); Probe, 200 nM: TgTTCATCACCTCTTC-MGBNFQ (Applied Biosystems)]. Cycling conditions were reverse transcription at 50°C for 15 minutes, and denaturation at 95°C for 5 minutes; then 45 cycles of 95°C for 1 second, 60°C for 20 seconds, followed by a single acquisition; and a final cooling step of 40°C for 30 seconds. Absolute quantification was compared to a viral RNA standard using LC480 software (version 1.5.0.39) and a standard calibrator on each plate. We present viremia data as PFU equivalents/mL using a 10∶1 PCR genome equivalent:PFU ratio that has been previously determined [29] and validated on human LASV samples.

### Validation of gene expression by RT-PCR

RT-PCR assays were carried out to quality check and validate our findings on the DNA microarrays. RT^2^ Profiler PCR Array from QIAGEN was used to run the RT-PCR. 125 genes of interest were plated on the custom array along with control genes. RNA extracted from the PBMCs (as described under RNA Processing and DNA Microarrays) was used for RT-PCR Array. RNA samples collected at three different time points from each animal were run on one plate. In all, six custom plates (each with a copy of the same probes for 125 genes) with samples from six different animals were run in this experiment. The RT-PCR experiment was performed as directed by RT^2^ RNA QC PCR Array Handbook 2012 (QIAGEN). Plates were then run on an ABI 7900 HT qPCR system (10 minutes at 95°C, 15 seconds at 95°C followed by 1 minute at 60°C×60 cycles). Following the PCR run for all 6 plates, the threshold was made uniform to be consistent among all the plates. Ct values for each sample were obtained. Results were interpreted using SDS software version 2.4 and data analysis software from SA Biosciences. Finally, data from different animals on a given day were pooled together and averaged. Data were then presented as fold change over day −8 expressions (Figure S4).

## Results

### Confirmation of an aerosol model of LASV infection

Prior studies in NHPs have described the course of LASV infection. However, most of these models have focused on disease caused by intramuscular (IM) injection. Only one study used aerosol model of LASV exposure [7]. Because human Lassa infection is likely caused by aerosol contact, we were interested in studying the immune response to infection in an aerosol model of exposure. To determine whether the disease progression following exposure to Lassa is similar to studies that carried out exposure to Lassa via IM injection, a pilot confirmation of virulence study was performed in which four NHPs were exposed to LASV via aerosol. At the target dose of 1,000 PFU, the actual dose each animal received ranged from 200 to 300 PFU (Figure S1A). Exposed animals all showed signs of disease and succumbed by day 16 post-exposure, with a mean time-to-death of 14.5 days (Figure S1B). All four NHPs showed increased levels (2–9 fold) of aspartate transaminase (AST) and showed signs of anorexia onset and recumbency at late times (day 11 onwards). These observations and their onset are consistent with low-dose intramuscular LASV challenges in cynomolgus macaques [4]. Animals experienced neurological signs to include seizures (three of four NHP) (Figure S1C).

### Transcriptome analysis of LASV exposure

Following this confirmation that LASV infection via aerosol exposure led to similar expected clinical signs and disease course development when compared to previous studies, we were interested in determining how circulating immune cells responded to LASV infection. Thus, we participated in a larger study analyzing multiple parameters of infection by sequential sampling of LASV exposed animals throughout the course of disease. A subset of animals was euthanized at different times post-exposure to understand the temporal progression of disease following LASV exposure. From this study, we obtained samples from eleven monkeys (represented by letters A–K in Figure 1A) that had been exposed to LASV (Josiah strain) via the aerosol route (target 1000 PFU). Prior to challenge, samples were taken for use as pre-exposure baseline controls (eleven samples at day −8).

The samples we obtained were circulating immune cells. At increasing times post-exposure, blood samples were collected and PBMCs were prepared from whole blood. Figure 1A illustrates the distribution of samples in the study. In the table, samples are sorted by days post-exposure. Corresponding clinical observations, viremia, and chemistry data that was also collected is briefly summarized in Figure 1B. The clinical data highlight that there were few signs of disease until 6–8 dpe, when viremia began and increased AST levels began to be observable. Clinical indications as well as severity increased throughout the disease course, with signs of anorexia and initial neurological signs appearing around 10 dpe. Based on this data, we conceptually divided the disease course into three separate stages, early (pre-symptomatic, 2 to <6 dpe), middle (early symptomatic, 6 to <10 dpe), and late (terminal disease, 10 to 12 dpe). These stages and their duration are pictorially illustrated in Figure 1C to highlight their correlation with asymptomatic disease (early), early symptomatic disease (middle), and increasing signs of disease (late).

### Global change in the gene expression in LASV-exposed NHPs

Overall, 30 PBMC samples were processed and hybridized onto DNA microarrays. This resulted in the generation of more than 1.3 million data points for analysis. Results from all arrays were computationally analyzed using the Limma software package in Bioconductor, a suite of packages in R. Our initial analysis focused on determining the major changes in mRNA expression over the course of LASV disease. This analysis showed that when experimental arrays were compared to the pre-exposure controls, more than 2,000 genes showed at least a 1.5 log2-fold change in their expression pattern in at least three arrays (Figure S2). These genes fall into categories such as immune response (153 genes), defense response (142 genes), response to wounding (113 genes), and inflammatory response (83 genes), each with a *p*-value <0.00001. Out of the 2,000 differentially expressed genes, 26.7% were downregulated and 73.3% were upregulated. These genes clustered into several different patterns that were particularly evident when arrays were grouped into categories of early, middle and late disease, similar to the arrangement of Figure 1A. The patterns observed did not appear to be due to the contribution of any one animal, as the removal of multiple arrays followed by re-clustering did not change the patterns observed (data not shown).

**Figure 2:**
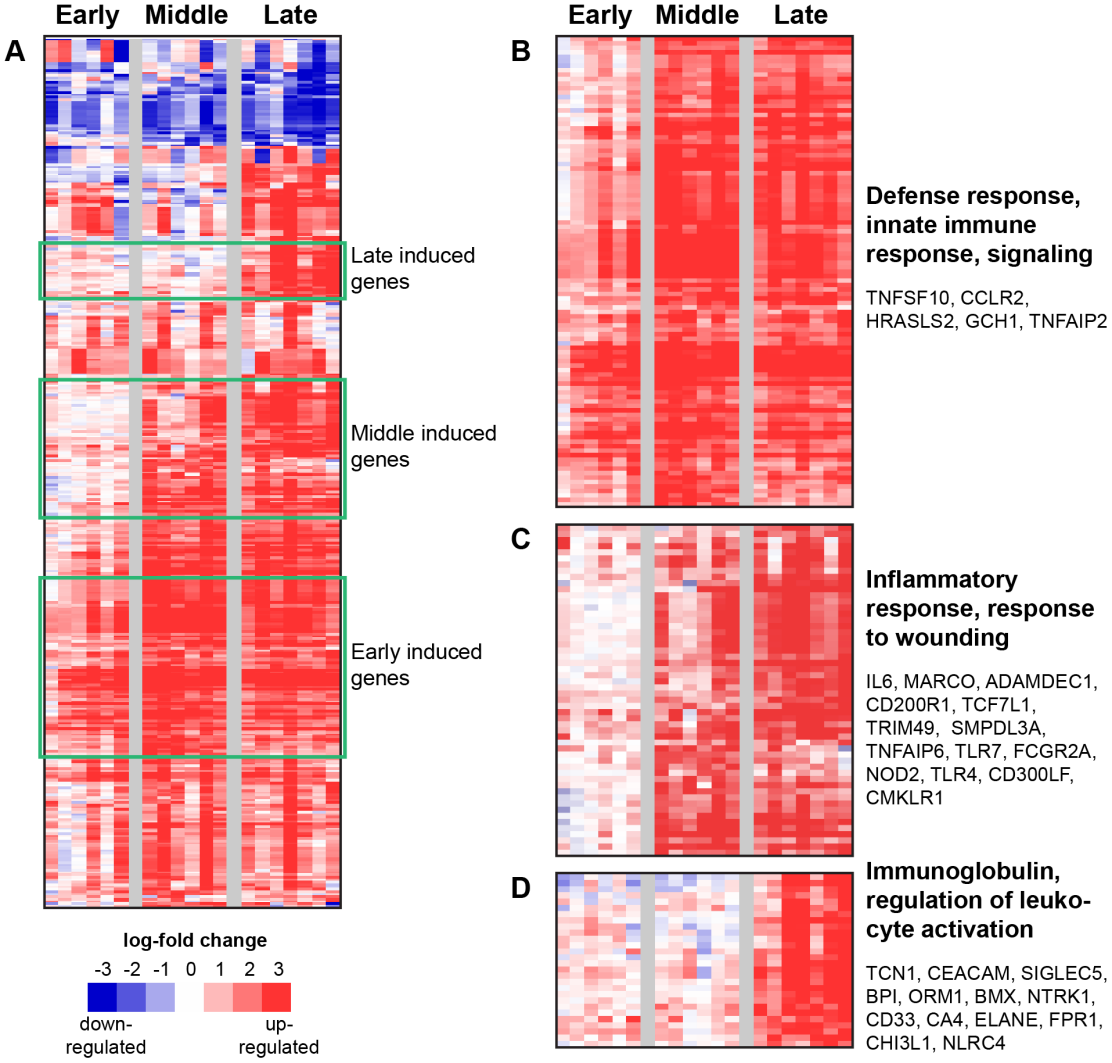
Strongly upregulated genes in LASV-exposed NHP. (A) Data had been zero-transformed using the pre-exposure control sample from each individual monkey to normalize for animal-intrinsic signatures and establish a baseline. Data were then filtered to identify 361 genes that showed at least a 2.5 log2-fold differential expression, and then hierarchically clustered. Each row in the heatmaps represents data from an individual gene, and each column represents the individual PBMC sample taken at a specific time point. Samples from the dataset were grouped into early (days 2–3), middle (days 6–8) and late (days 10–12) disease categories based on the day the sample was collected post-viral challenge. Red and blue colors denote expression levels greater or less than baseline (white), respectively. Green boxes identify significant clusters of genes induced during early, middle, and late disease, and are labeled accordingly. An expanded view of these gene clusters is shown in (B), (C), and (D). The most significant functional groups (assigned by DAVID, *p*-value<0.001) found in the respective clusters are listed to the right of the heatmaps, along with the names of some representative genes.

From this set of 2,000 genes that were significantly regulated following LASV exposure, we were particularly interested in the most strongly regulated genes. Figure 2A shows a clustered heatmap of probes that showed a fold-change of greater than 2.5 log2 (>5-fold change) following exposure. Within these highly regulated probes, three major types of regulation are readily visible in the clustered image. Probes contained in the upper green-boxed region (expanded in Figure 2D) showed little change expression early in infection but increased expression late in infection. Probes in the middle box (expanded in Figure 2C) showed upregulation at middle and late times post-exposure. Probes in the lower box (also Figure 2B) showed strong expression early post-exposure that was maintained throughout the course of the study. Early induced genes were associated with the innate immune response by gene ontology analysis. Genes induced in the middle of the disease course were mRNAs associated with the inflammatory response. Genes strongly induced late post-exposure (Figure 2D) were associated with cell cycle regulation, cell division, and DNA packaging. To assess the reproducibility of these findings a number of up and down-regulated genes were assayed by rtPCR (Figure S4). The selected genes validated well, illustrating that the answers derived from our array analysis are transferrable to a PCR-based detection format.

### LASV exposure results in the early upregulation of immune response genes

We noted that probes associated with genes involved in the innate immune response markers such as TRAIL (TNFSF10), MX1, and CCRL2 demonstrated a rapid innate response that was evident before there are clinical signs of disease (Figure 2B). Early induced genes also included IRF3-responsive genes such as phospholipase A2 gamma, OAS1/2, and GBP1 [30]. Additionally, we observed that GCH1 showed rapid upregulation. GCH1 is the rate-limiting enzyme regulating the synthesis of pain hormone BH4 and increased GCH1 is associated with increased BH4 levels. Patients with LF often complain of increased pain [31].

Probes that recognized the IL6 gene dominated the list of probes that were upregulated in the middle of the disease course (Figure 2C). IL6 showed little to no induction at early times post exposure but was strongly upregulated by 6 dpe. Several pattern recognition receptors like MARCO, TLR4, TLR7, NOD2, and Fc receptor FCGR2A showed a similar time course of transcriptional activation, as do inhibitory receptors for macrophages function like CMKLR1, CD200R1, and CD300LF. Basophil activation marker CD63 was also upregulated beginning around 6 dpe, suggesting the activation of these cells. There was also transcriptional evidence for significant immune cell movement during this stage of LASV disease. At 6 dpe, chemotactic markers such as CCR5, CCL23, CXCL12, and TNFAIP6 were upregulated. This coincident upregulation of innate immune sensors and responses along with repressors of adaptive responses in the form of inhibitor receptors suggests a skewing of the immune response towards the innate.

Genes that were upregulated at late times in the LASV disease course (Figure 2D) included a large number of granulocyte markers such as TCN1, CEACAM molecules, and SIGLEC5. Transcripts of exocytic granule components such as BPI, ELANE and the neutrophil chemotaxis promoting receptor FPR1, are also upregulated late in response to LASV exposure. Analysis of the cell composition of the blood in the LASV-exposed NHPs at different times post-exposure also suggests the predominance of neutrophils at late times post-exposure (Figure S3). In addition to upregulation of neutrophil-specific transcripts, we also detected upregulation of immunomodulatory proteins at late times post-exposure: ORM1, tyrosine kinase genes like BMX and NTRK1, myeloid specific siglec3 (CD33), and enzymes such as CHI3L1, carbonic anhydrase (CA4), and the apoptosis regulator, NLRC4.

### Strongly upregulated genes implicate PAMP receptors, IRFs, and metalloproteinases as significant components of the immune response to LASV

**Figure 3:**
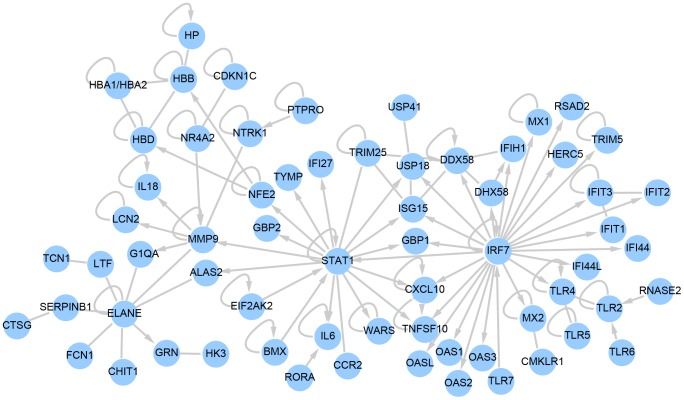
A signaling networks of 65 genes which are strongly upregulated following LASV challenge of NHP. Ingenuity Pathway Analysis software was used to analyze a list of 360 genes which were found to be strongly upregulated following LASV challenge. Of this list, 65 were found to be directly or indirectly connected according to Ingenuity's database of published interactions. Nodes represent individual gene products, with official gene symbols included. Edges without arrows indicate a direct interaction between two gene products, *e.g.* a protein-protein interaction. Edges with arrows represent a regulation of transcription, directed from the gene regulator to a regulated gene. Loops indicate self-regulation. Notable in the expression profiles is the upregulation of STAT1 and IRF7, MMP9 and ELANE signaling nodes.

When the strongly upregulated genes from Figure 2A were analyzed using Ingenuity Pathway Analysis (IPA), 65 were observed to be connected through transcriptional or protein-protein interactions (Figure 3). As was expected from the identification of many innate immune stimulated genes in the early induced population of transcripts, our analysis identified two transcriptional nodes that are upregulated in response to LASV exposure: (1) IRF3/IRF7 induced genes such as OASs, IFITs, MX1, and TRIM5; and (2) STAT1 induced genes such as ISG15, TRIM25, GBP2, TRAIL, and PKR (EIF2AK2). The identification of both types of genes suggests that the circulating immune cells are generating a strong innate immune response very early post-LASV exposure. Interestingly, there was not a strong transcriptional upregulated interferon α, β or γ in PBMCs, despite the expectation that these genes would be strongly upregulated due to the potential signaling through STAT1.

The identification of IRF3/IRF7 responsive genes in LASV exposure suggested that there was some signaling through either Toll-like receptor (TLR) or RIGI-like receptor (RLR). Consistent with this, we saw significant (more than 1.5 log2 fold) increases levels of several TLR receptors (TLR1 through TLR7) induced at early times post-exposure (Figure S5). Of the upregulated TLRs, transcripts of TLR3, TLR4, TLR5, TLR6, and TLR7 were the most strongly expressed. Along with TLR upregulation, we also saw significant increase in the transcripts of RLR genes such as DDX58 (RIGI) and DHX58 (LGP2) at early times post-exposure. The coordinated upregulation of these genes strongly indicates that during challenge with LASV the infected host increases its ability to respond through these pathways.

IPA analysis also identified a collection of genes that are associated with neutrophil granules. These include three proteases that are upregulated during the course of infection: the matrix metalloprotease MMP9, the serine proteases neutrophil elastase (ELANE), and cathepsin G (CTSG), as well as the protease inhibitor SERPINB1. These proteases have important antimicrobial properties, as do other neutrophil granule proteins increased during LASV infections such as lactotransferrin (LTF) and lipocalin 2 (LCN2). This correlates with the increase in the neutrophils seen at late times post-exposure (percentage and counts, Figure S3) in the peripheral blood of LASV-exposed NHPs. This coordinated upregulation of multiple neutrophil granule proteins in the PBMC samples may reflect accelerated granulopoiesis and the mobilization of neutrophil precursors into the blood, consistent with a neutrophillic response to severe LASV infection. Interestingly, protease upregulation has been noted in other animal models of filovirus infection [32].

### Analysis of cytokine and chemokine expression following LASV exposure

**Figure 4:**
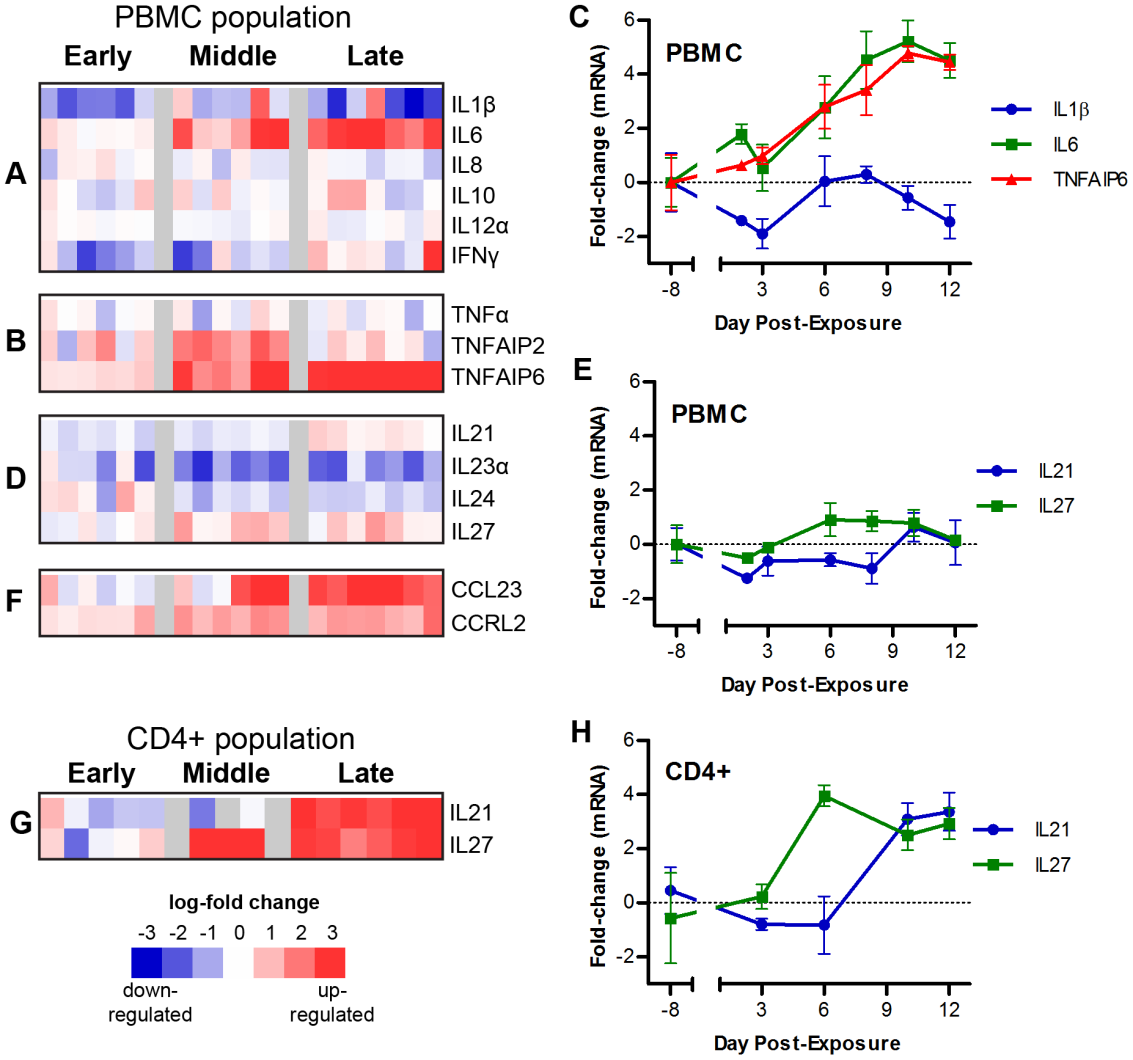
Cytokine mRNA expression following LASV challenge. Heatmaps show the gene expression levels of (A) previously analyzed cytokines in LASV disease: IL1β, IL6, IL8, IL10, IL12α, and IFNγ; (B) TNFα and TNF-responsive genes; (D) cytokines IL21, IL23α, IL24, and IL27; and (F) chemokines CCL23 and CCRL2 from the whole PBMC population; as well as (G) IL21 and IL27 from the CD4+ population (isolated from PBMCs). Line graphs show the expression levels (mRNA log2 fold change over time) of (C) IL1β, IL6, and TNFAIP6; and (E) IL21 and IL27 from the whole PBMC population; and (H) IL21 and IL27 in the CD4+ population.

Moving beyond the analysis of highly differentially expressed genes, we were interested in how the gene expression changes in our arrays compared with changes already noted in animal and human models of disease; therefore, we analyzed the expression of IL1β, IL8, IL10, IL6, IL12, IFNγ, and TNFα. Earlier reports have followed the expression of these cytokines in LASV-infected animals and humans [4], [21], [33], [34] and have shown that plasma levels of IL8, IL10, IL6, IL10, TNFα, and IFNγ increase with LASV infection. IL1β was induced very late (day 13 onwards). Our data show that IL1β transcripts are down-modulated following LASV exposure (Figure 4A and 4C). This suppression was clear at early times post-exposure (animals showing a 1–2 log2 fold decrease) and persisted throughout the disease course with some variability between animals. Transcripts of IL6 were upregulated during the middle stage of disease, from 6 dpe (Figure 4A and 4C). Transcripts of IL8 were not differentially expressed in PBMCs at any stage (early, middle or late) of LASV disease. Similarly, IL10 transcripts were not differentially expressed in the PBMCs of LASV-exposed NHPs, nor were IL12 transcripts. These results are in line with earlier reported studies on fatal cases of LASV patients [33]. Transcripts of IFNγ were down, and transcripts TNFα were not differentially expressed in our model of LASV infection; however, during the middle stage of LASV disease, there was an upregulation of the TNFα-induced genes, TNFAIP2 and TNFAIP6 (Figure 4B and 4C). This finding correlates well with an earlier study on human patients of LF [35].

In addition to this data showing concordance of our data with previous studies, we also saw differential expression of cytokines that have not been previously reported in the context of LASV infection. Transcripts of IL21 were slightly upregulated (1–1.5 log2 fold) in PBMCs at 10 dpe (Figure 4D and 4E). Transcripts of IL21 were strongly upregulated (more than 3 log2 fold) in the separated CD4 positive cells (Figure 4G and 4H). Upregulation of IL21 has been reported in LCMV model of HF and has been linked to the clearance of infection but had not previously been noted in LASV infection [36]. Transcripts of IL23α and IL24 were down-modulated (2–1.5 log2 fold) at 3 dpe and 6 dpe respectively, during LASV exposure (Figure 4D). Interestingly, we detected upregulation (1.5 log2 fold) at 6 dpe of transcripts of an immunosuppressive cytokine, IL27 (Figure 4D and 4E). This upregulation of IL27 was more pronounced (4 log2 fold) at 6 dpe in CD4 positive cells than in the PBMC population (Figure 4G and 4H). IT is possible that IL27 protein may play an important function in the response to LASV infection.

In addition to these changes in cytokine expression, chemokines such as CCRL2 and CCL23 are also upregulated in the LASV-exposed NHPs. CCRL2, a marker of monocytic in-filtration in inflammatory diseases [37], is significantly upregulated (1.5–2 log2 fold) early (3 dpe) following LASV exposure, and CCL23 [also known as macrophage inflammatory protein 3 (MIP3)], a potent chemoattractant for T lymphocytes and monocytesis, is upregulated (more than 3 log2 fold) at middle times post-exposure (8 dpe) (Figure 4F). Together, the upregulation of CCRL2 and CCL23 suggests that LASV exposure causes an increase in the call for the recruitment of inflammatory cells.

### Innate immune gene expression in circulating immune cells precedes the detection of viremia in LASV exposed NHPs

**Figure 5:**
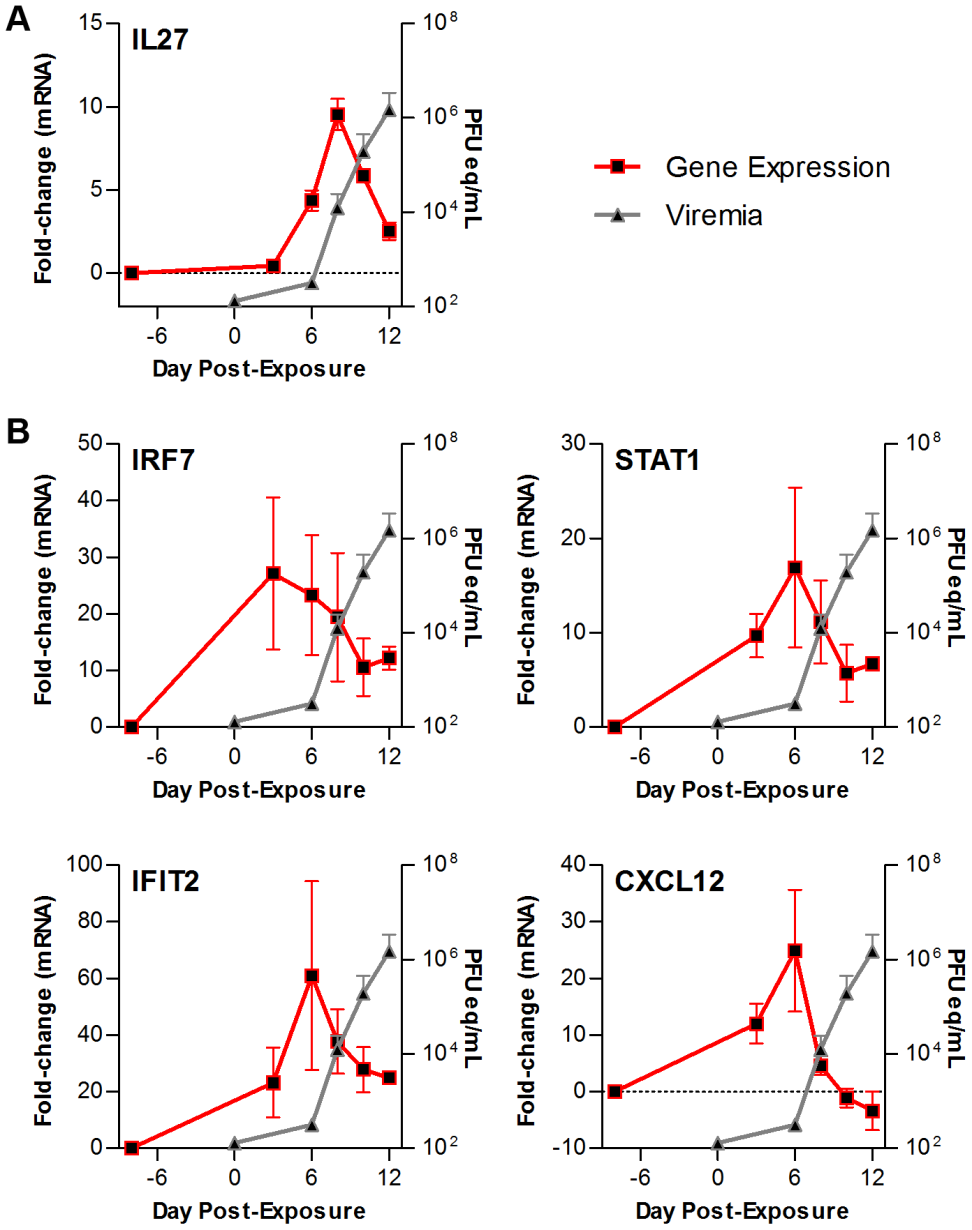
Gene expression precedes the detection of viremia in LASV-exposed NHPs. Comparison of observed mRNA expression level changes over the course of infection (red line) to the appearance of virus in the blood (grey line) in LASV-exposed NHPs. Shown here are probes for (A) the cytokine IL27; and (B) genes involved in the innate antiviral response: IRF7 (top left), STAT1 (top right), IFIT2 (bottom left), and CXCL12 (bottom right). Error bars represent S.E.

To understand the relation between the timing of transcript upregulation in PBMCs and the onset of viremia in LASV disease, viral load was evaluated throughout the course of infection by RT-PCR. Transcripts a selected set of upregulated genes were compared to the viral load in the plasma at different time points in Figure 5. Comparisons in Figure 5A highlights that while viremia was not observed until 8 dpe, transcripts of cytokine IL27 showed increased expression earlier (approximately 6 dpe). More dramatically, innate antiviral response molecules such as IRF7, STAT1, and IFIT2, and chemokines such as CXCL12 are upregulated by 3 dpe (Figure 5B). This demonstrates that the expression of host transcripts (immune response) in response to LASV exposure significantly precedes the onset of circulating viremia in infected NHPs.

**Figure 6:**
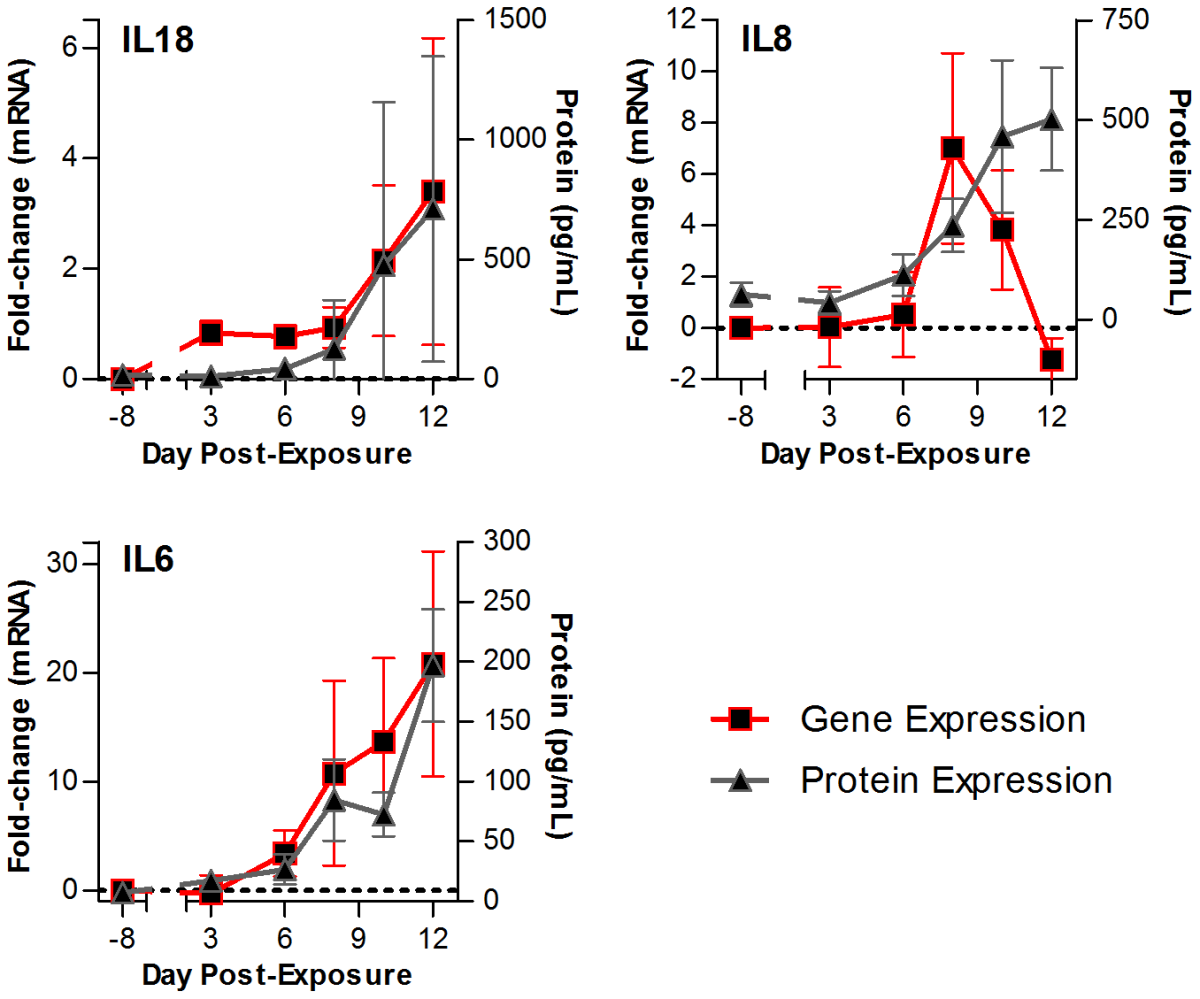
Comparison of the cytokine gene expression with the cytokine levels in the plasma of LASV-exposed NHPs. Line graph to show the comparison of gene expression (red line) to the amount of cytokine detected (in pg/mL, grey line) in the plasma of LASV-exposed NHPs over the course of the disease. Shown here are probes for IL18 (top left panel), IL8 (top right panel), and IL6 (bottom left panel). Error bars represent S.E.

We also compared the cytokine gene expression to the cytokine protein expression seen in the plasma of LASV exposed NHPs (Figure 6). Cytokine levels in blood samples were determined prior to LASV exposure and throughout the course of disease. This comparison of gene expression with the actual protein levels of these cytokines (Figure 6) revealed that the increased gene expression of a subset of cytokines correlated with protein expression (e.g. IL8, IL6 and IL18). However, this correlation was not always observed. For IL8, an initial correlation of mRNA and protein expression at early times broke down by 12 dpe, where we observed a loss of mRNA expression but protein expression was still readily detectable. This could be due to either loss of the IL8 producing cell population from the PBMCs due to migration or cell death, or it is also possible that another cell population accounts for the observed protein in the plasma at late times of disease. We also observed that array analysis correctly characterized proteins that were expressed in response to viral exposure, such as IL1β, IL12, and IL10 (data not shown).

### Immunosuppressive genes are induced following LASV exposure

As LASV disease in humans is associated with an inefficient host immune response to infection [38], we determined the expression level of several immune suppressive genes (negative regulators of immune responses) with the hypothesis that PBMCs would upregulate immunosuppressive genes would be upregulated in PBMCs in response to LASV exposure. Table 1 describes the upregulation of genes involved in regulating T cell function, such as PDL1 (CD274) [39] and TNFRSF21 [40], that are upregulated within the first 6 dpe. We also see upregulation of the regulatory players that are involved in intracellular signaling and have been linked to immunosuppressive function. For example, transcripts of the following were upregulated during LASV disease: ITIM domain containing receptor LILRB2, an adapter protein DOK3 [41], [42], an acute phase protein ORM1 [43], an ADP ribosylation factor PARP14 [44], a phosphogluconate dehydrogenase PGD [45], a translational repressor SAMD4A, a neutrophil elastase inhibitor SERPINB1 [46], and TGFβ induced protein TGFBI. TGFBI can cause decrease the p53 binding to DNA leading to down-modulation of p53 mediated genes (DR5, JUNB, TET2, and GADD45, as seen in our gene expression data). TGFβ has been reported in the plasma of LCMV-infected rhesus macaques [34].

**Table 1:** Expression of genes that regulate immunological response following LASV exposure.

Negative Regulators of Immune Response			
	EARLY	MIDDLE	LATE
CD274	0.4	1.8	1.9
TNFRSF21	0.3	2	1.2
TGFBI	0.6	1	−0.7
DOK3	0.5	1	1
SERPINB1	0.3	2	2.4
LILRB2	0.7	1.5	1.1
SAMD4A	1.2	3.3	2.3
ORM1	−0.1	−0.1	3.3
PGD	−0.2	0.9	1.6

Positive Regulators of Immune Response			
	EARLY	MIDDLE	LATE
JUNB	−0.6	−0.3	−0.6
FOSB	−1.4	−1	−1.9
CD69	−1.6	−2	−2.1
CD83	−1.3	−1.3	−1.4
STAT4	−0.4	−0.8	−0.9
GADD45A	−0.7	−1	0.8
SIGLEC10	0.5	−1.1	−1.5

Table showing the log2 fold change (from pre-exposure control) in the expression of the negative or positive regulators of immune-response genes at early, middle, and late induced times post-LASV infection of NHPs.

Along with upregulation of these immunosuppressive genes, there was a significant early down-modulation of positive immune response regulating genes such as JUN, FOS, CD69, CD83, STAT4, GADD45A, and SIGLEC10 in PBMC from LASV exposed NHPs (as shown in Table 1), suggesting the lack of key players in the generation of immune response to infection. Expression of these genes is shown as a heatmap and line graphs in Figure S6. Together, the behavior of these genes is consistent with an upregulation of immunosuppressive signals, compounded by the lack of factors that lead to the development of adaptive immune response such as production of IL2 and IL4 following virus infection.

## Discussion

At the outset of this study, it was unclear whether the PBMC population would provide an accurate report of the systemic immune response; however, our analysis has proven that these cells appear to respond to LASV exposure in ways that closely mirror what has already been described for the response to infection in humans and NHP [4], [21], [33], [47]. These changes include the lack of IL1β, IL8, TNFα, IL2, IFNγ, IL4, IL12, and IL10, and the upregulation of IL6. In our system, we see an induction of IL6, consistent with earlier descriptions of LASV associating this cytokine with fatal cases of human patients [48] as well as animal models [4].

Our data show that there is both an early induction as well as sustained activation of IRF3/IRF7 responsive genes following aerosol exposure to LASV (Figure 2). This robust response was evident very early post-exposure prior to onset of clinical signs or detection of viremia and was maintained throughout the disease course. This potent innate response does not appear to be coupled to a strong adaptive immune response, as IL4, IFNγ, and IL2 were not upregulated in the PBMC at any point in response to LASV exposure. One suggestion for why there is a poor adaptive response is that immunosuppressive responses are dominating the PBMC response to exposure. Our data shows that there is strong expression of genes which can have interferon-suppressive activity, such as TGFBI [49], CD274 (PDL1), TNFRSF21, and IL6 [50]. This is consistent with the hypothesis that the overwhelming innate antiviral response seen in LASV-exposed macaques is compromising the downstream immune response by either inhibiting DC differentiation [51] or cytokine responses [52], or causing attrition of T cells (mainly CD8+ cells) [53], [54].

We also found that during LASV disease there is increased expression of IL27 and IL21 mRNAs. Upregulation of IL21 was previously noted in a LCMV model of hemorrhagic fever [36] and recent studies have provided genetic evidence that the IL21 gene is important for successful responses to LASV exposure [55], suggesting that further analysis of the importance of IL21 in LASV exposure is warranted. Our finding that IL27 transcripts are upregulated early in LASV exposure is interesting because IL27 enhances the expression of the immunosuppressive PDL1 gene [56] and inhibits T cell function. Thus, increased IL27 provides a potential mechanism for the observed suppression of adaptive cytokines such as IL2 and IL4.

Comparison of our results with other microarray-based analyses of arenavirus infection shows that our data correlate well with other models of arenavirus-induced hemorrhagic fever [34]. Both studies found similar changes in the STAT1/interferon responsive genes such as TRAIL, IFI44, OASs, IFIT1, and IFIT2 showing a strong innate immune response. Our data suggest that the innate response is slightly more rapid in LASV than in LCMV infected primates. Both studies show similar changes in the down-modulation of immune response genes such as IL1β, CXCR4, IL8, and IL24 and the activation of transcription factors STAT1, STAT2, SOX4, NR4A2, SMAD7, and TRIM25. Thus, comparison of our study with LCMV infection model suggests that arenavirus infection can lead to highly similar transcriptional fingerprints, which have a few notable differences in differing kinetics of interferon induction and expression of CCL5 and CREB1.

The similarity of the transcriptional response in the two arenavirus models of hemorrhagic fever is in stark contrast to that seen in Ebola virus (EBOV)-infected animals. In both EBOV- and LASV-exposed animals, there is a strong induction of IRF3 and STAT1-responsive genes [24], [57], but the response following LASV exposure appears to be earlier than that seen for EBOV-infected monkeys. Also, a lack of IL1β and low levels of IL8 are seen in the LASV model, but these genes are strongly expressed in cells from EBOV-exposed NHPs [24], [57]. PBMCs from LASV exposed animals do not express the apoptotic and clotting factor genes associated with EBOV disease, further highlighting the fundamental differences in the immune response to these different infections.

Our studies suggest that tracking gene expression in circulating immune cells is a worthwhile avenue to explore for early disease diagnosis. Comparison of cytokine gene expression with the amount of detectable protein in LASV-exposed NHPs demonstrated a positive correlation between the gene expression and protein expression for a subset of cytokines such as IL6, IL8, and IL18, suggesting no diagnostic or prognostic advantage for transcript-based analysis. In contrast, our microarray system provided the ability to identify major changes in interferon-stimulated genes considerably before the onset of viremia in LASV exposed NHPs (Figure 5), suggesting that analyzing the expression of these transcripts or proteins could provide pre-symptomatic detection of disease. This would be directly analogous to recent work showing that pre-symptomatic detection of influenza is possible [58] but additional analysis will be necessary to fully assess the possibility.

To our knowledge, this is the first whole-transcriptomics analysis of the response of the primate circulating immune system to LASV exposure. This analysis validates the underlying model as a faithful reproduction of the human disease and demonstrates that much of the immune response can be tracked through analysis of the circulating immune cells. Our results highlight that a transcriptomics approach allows the analysis of previously investigated cytokines such as IL1β, IL8, and IL6. Additionally, it allows the simultaneous analysis of additional genes that this study has found are strongly upregulated, such as IL27 and CD274 (PDL1). Our data also shows that the circulating immune response shows a strong innate response to infection that is visible hours to days before the earliest clinical assay (viremia) identifies infection in this model, suggesting that future studies may be able to capitalize on this information to develop pre-symptomatic diagnostics for LASV infection.

## Supporting Information

Figure S1 **Confirmation of lethality of aerosol model of LASV.** (A) This table shows the actual dose of infectious particles received by the animal from the targeted 1000 PFU dose. (B) Kaplan-Meier survival curve for cynomolgus macaque confirmation of virulence study following aerosol exposure to LASV. (C) Clinical and pathological symptoms observed in LASV-exposed NHPs.
(TIF)

Figure S2 **Distinct sub-patterns in gene clusters reflect temporal expression.** (A) Data was zero-transformed using the pre-exposure control sample from each individual monkey to normalize for animal-intrinsic signatures and establish a baseline. Data were then filtered to identify over 2000 genes that showed at least a 1.5 log2-fold differential expression, and hierarchically clustered. Each row in the heatmaps represents data from an individual gene, and each column represents the individual PBMC sample taken at a specific time point. Samples from the dataset were grouped into early (days 1–3), middle (days 6–8) and late (days 10–12) disease categories based on day the sample was collected post-viral challenge. Major gene clusters are denoted by the colored vertical bars to the right of the heatmap and are labeled with the significant functional groups for that cluster as identified through DAVID. Significant gene clusters that appear to be expressed temporally during the early, middle, and late disease stages are indicated by green boxes, and are expanded upon in (B), (C), and (D), respectively. The most significant functional groups (assigned by DAVID, *p*-value<0.001) found in these clusters are listed to the right of the heatmaps, along with the names of some representative genes. Red and blue colors denote expression levels greater or less than baseline (white), respectively.
(TIF)

Figure S3 **Quantification of white blood cells (WBCs) in the peripheral blood of LASV exposed NHPs.** (A) A line graph showing the percentage of neutrophils (green line), lymphocytes (blue line), and monocytes (red line) in the blood of LASV-exposed NHPs. (B) A table showing the absolute numbers of neutrophils, lymphocytes, and monocytes in the blood of LASV-exposed NHPs at different times (day) post-exposure. Standard error is also listed next to each number.
(TIF)

Figure S4 **Real-time PCR validation of DNA microarray gene expression.** (A) Table shows the fold change in gene expression from day −8 either in a real-time PCR assay or DNA microarray. Genes are classified into three categories (early, middle, and late induced). (B) Bar graph comparing fold change in gene expression obtained by RT-PCR (blue bars) and DNA microarray (red bars). (C) Line graph showing fold expression of three representative genes (DDX58, CD63, and STAT2) over time by both RT-PCR assay (blue line) and DNA microarray (red line).
(TIF)

Figure S5 **Expression of Toll-like receptors (TLRs) and RIGI-like receptors (RLRs) in LASV-exposed NHPs.** (A) Heatmap of TLR1 through 7, DDX58, and DHX58 in the LASV-exposed macaques (DNA microarray). (B) Line graph showing the expression of TLR3 (blue line), TLR4 (green line), TLR5 (red line), and TLR7 (black line) in the PBMCs of LASV-exposed macaques by RT-PCR.
(TIF)

Figure S6 **Immunological response.** Heatmap of (A) negative and (B) positive regulators of immune response. Line graphs of representative genes from (C) negative regulators represented in A and (D) positive regulators of immune response represented in B, following LASV exposure.
(TIF)
