# Supplementary figures and images for: Transcriptional Profiling of the Circulating Immune Response to Lassa Virus in an Aerosol Model of Exposure
Source: PLoS Negl Trop Dis. 2013 Apr 25;7(4):e2171. doi: 10.1371/journal.pntd.0002171 (PMC3636129; doi:10.1371/journal.pntd.0002171)

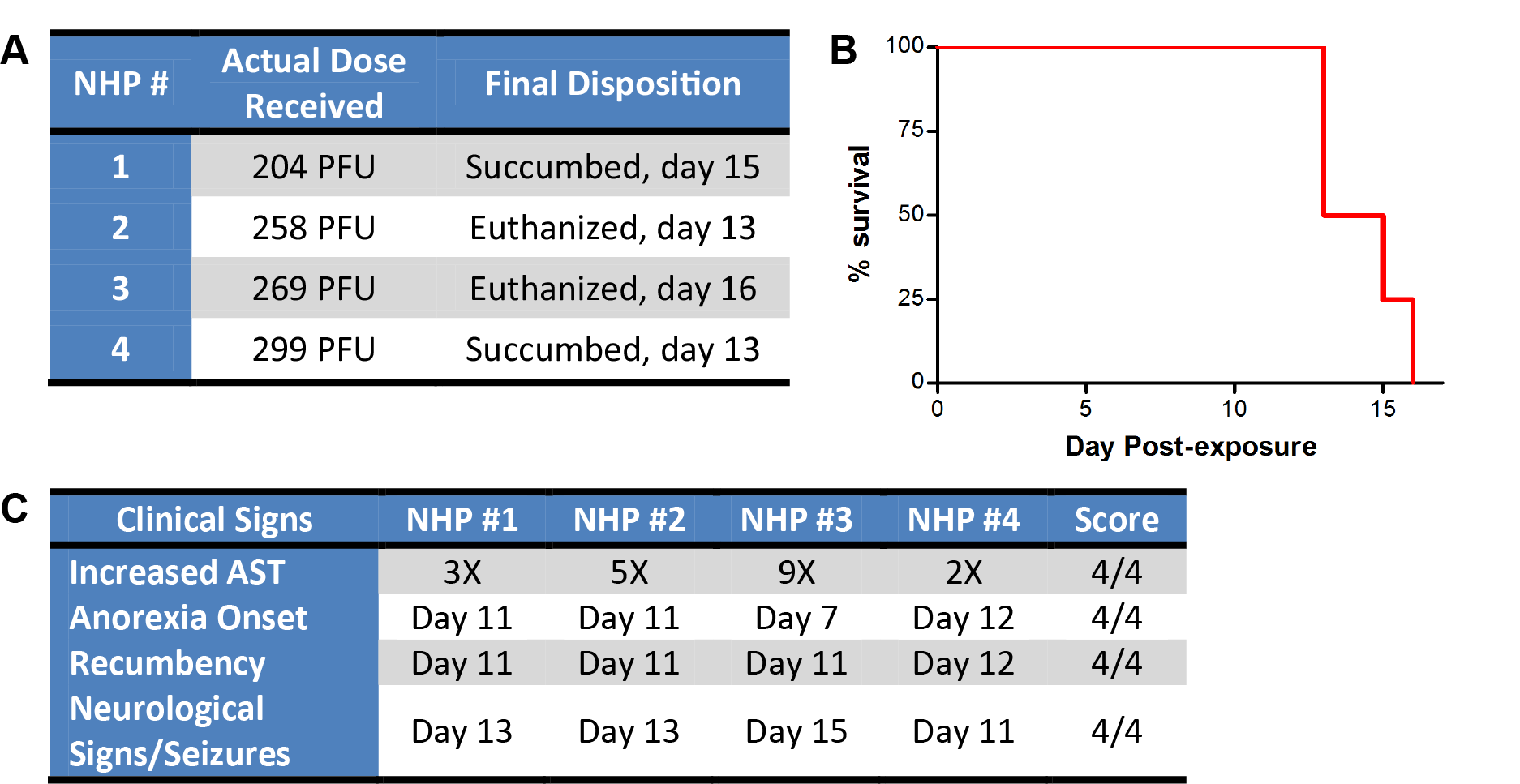

Supplement: Figure S1 — Confirmation of lethality of aerosol model of LASV. (A) This table shows the actual dose of infectious particles received by the animal from the targeted 1000 PFU dose. (B) Kaplan-Meier survival curve for cynomolgus macaque confirmation of virulence study following aerosol exposure to LASV. (C) Clinical and pathological symptoms observed in LASV-exposed NHPs. (TIF) [file pntd.0002171.s001.tif]

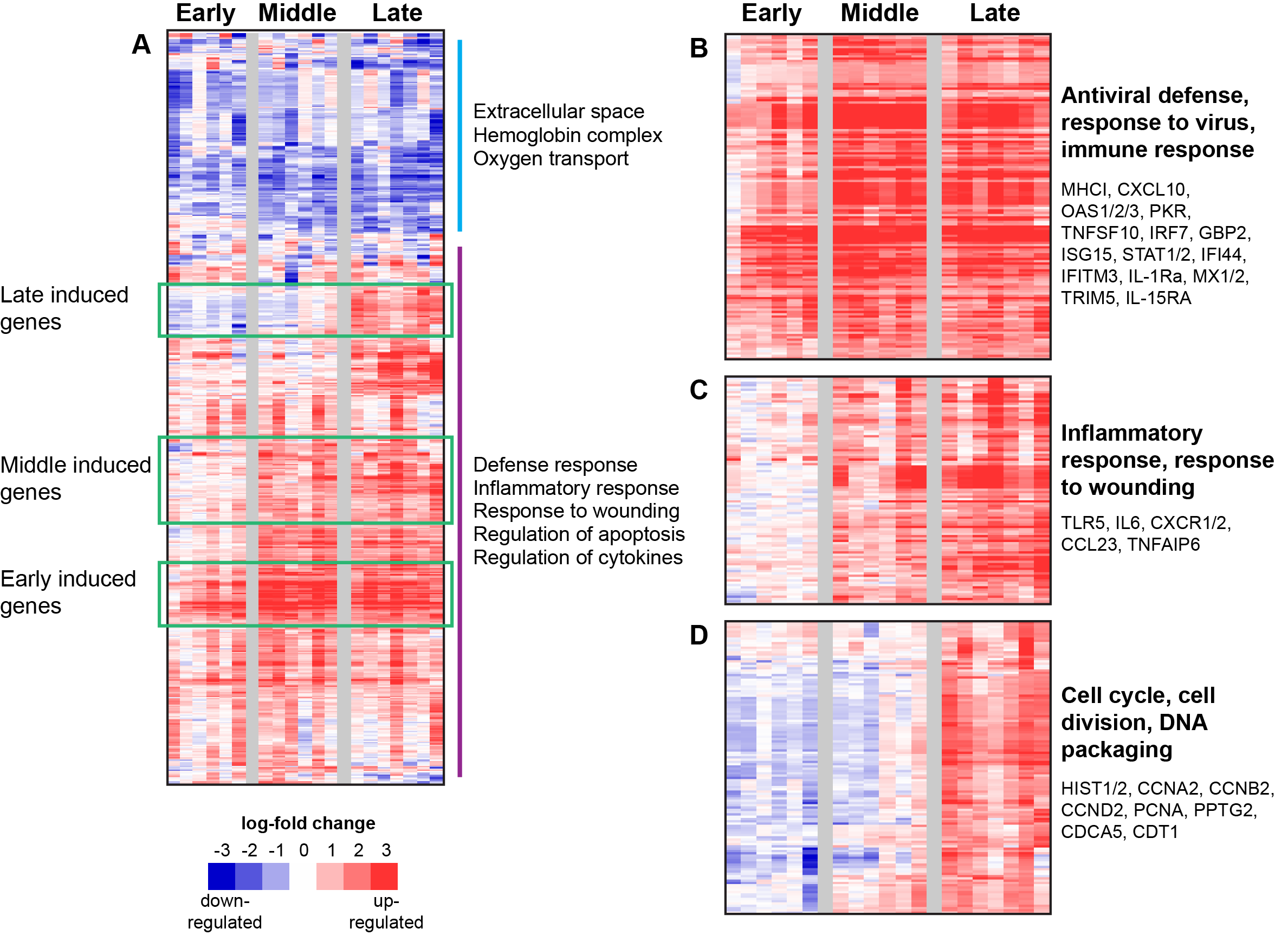

Supplement: Figure S2 — Distinct sub-patterns in gene clusters reflect temporal expression. (A) Data was zero-transformed using the pre-exposure control sample from each individual monkey to normalize for animal-intrinsic signatures and establish a baseline. Data were then filtered to identify over 2000 genes that showed at least a 1.5 log2-fold differential expression, and hierarchically clustered. Each row in the heatmaps represents data from an individual gene, and each column represents the individual PBMC sample taken at a specific time point. Samples from the dataset were grouped into early (days 1–3), middle (days 6–8) and late (days 10–12) disease categories based on day the sample was collected post-viral challenge. Major gene clusters are denoted by the colored vertical bars to the right of the heatmap and are labeled with the significant functional groups for that cluster as identified through DAVID. Significant gene clusters that appear to be expressed temporally during the early, middle, and late disease stages are indicated by green boxes, and are expanded upon in (B), (C), and (D), respectively. The most significant functional groups (assigned by DAVID, p-value<0.001) found in these clusters are listed to the right of the heatmaps, along with the names of some representative genes. Red and blue colors denote expression levels greater or less than baseline (white), respectively. (TIF) [file pntd.0002171.s002.tif]

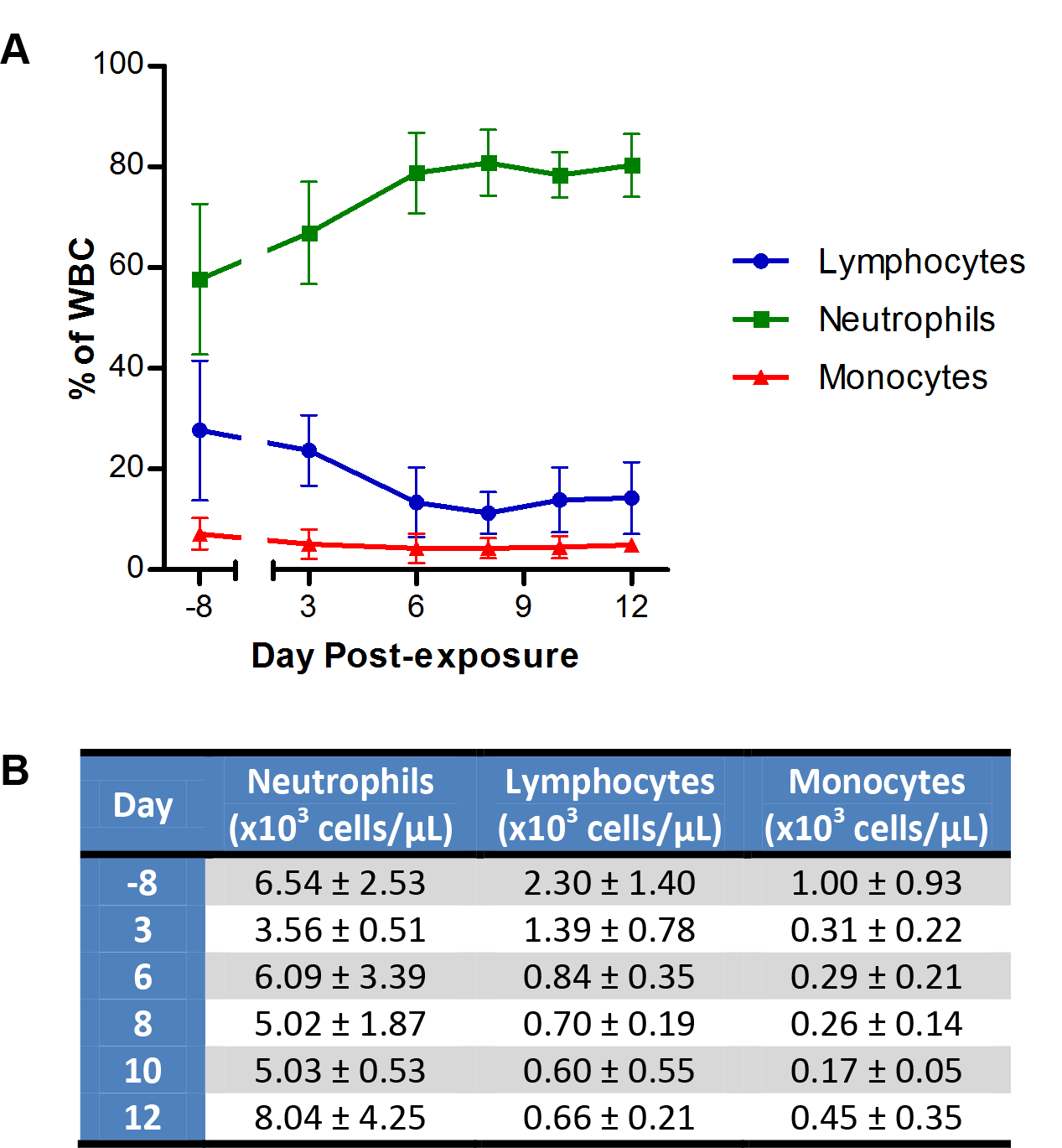

Supplement: Figure S3 — Quantification of white blood cells (WBCs) in the peripheral blood of LASV exposed NHPs. (A) A line graph showing the percentage of neutrophils (green line), lymphocytes (blue line), and monocytes (red line) in the blood of LASV-exposed NHPs. (B) A table showing the absolute numbers of neutrophils, lymphocytes, and monocytes in the blood of LASV-exposed NHPs at different times (day) post-exposure. Standard error is also listed next to each number. (TIF) [file pntd.0002171.s003.tif]

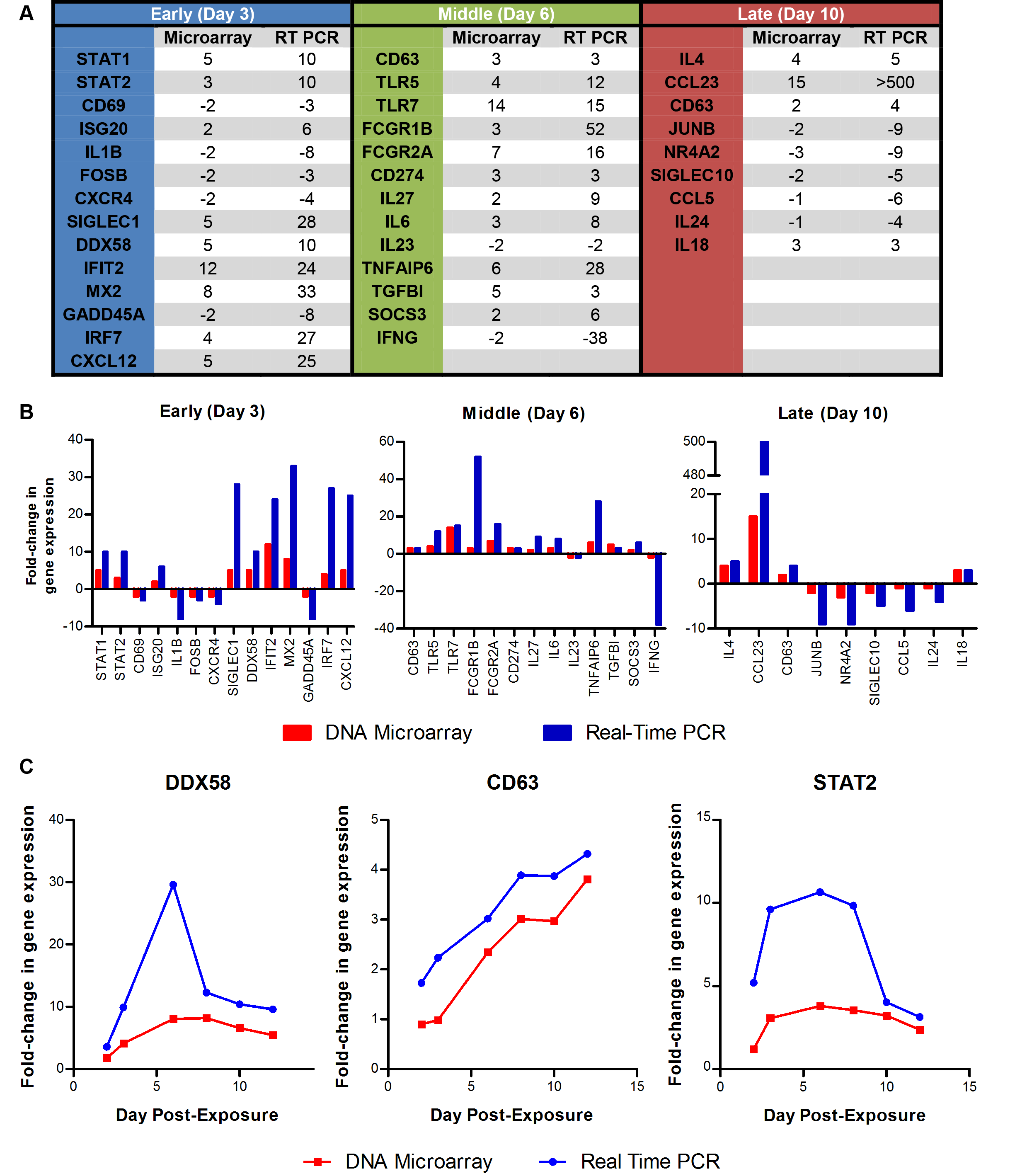

Supplement: Figure S4 — Real-time PCR validation of DNA microarray gene expression. (A) Table shows the fold change in gene expression from day −8 either in a real-time PCR assay or DNA microarray. Genes are classified into three categories (early, middle, and late induced). (B) Bar graph comparing fold change in gene expression obtained by RT-PCR (blue bars) and DNA microarray (red bars). (C) Line graph showing fold expression of three representative genes (DDX58, CD63, and STAT2) over time by both RT-PCR assay (blue line) and DNA microarray (red line). (TIF) [file pntd.0002171.s004.tif]

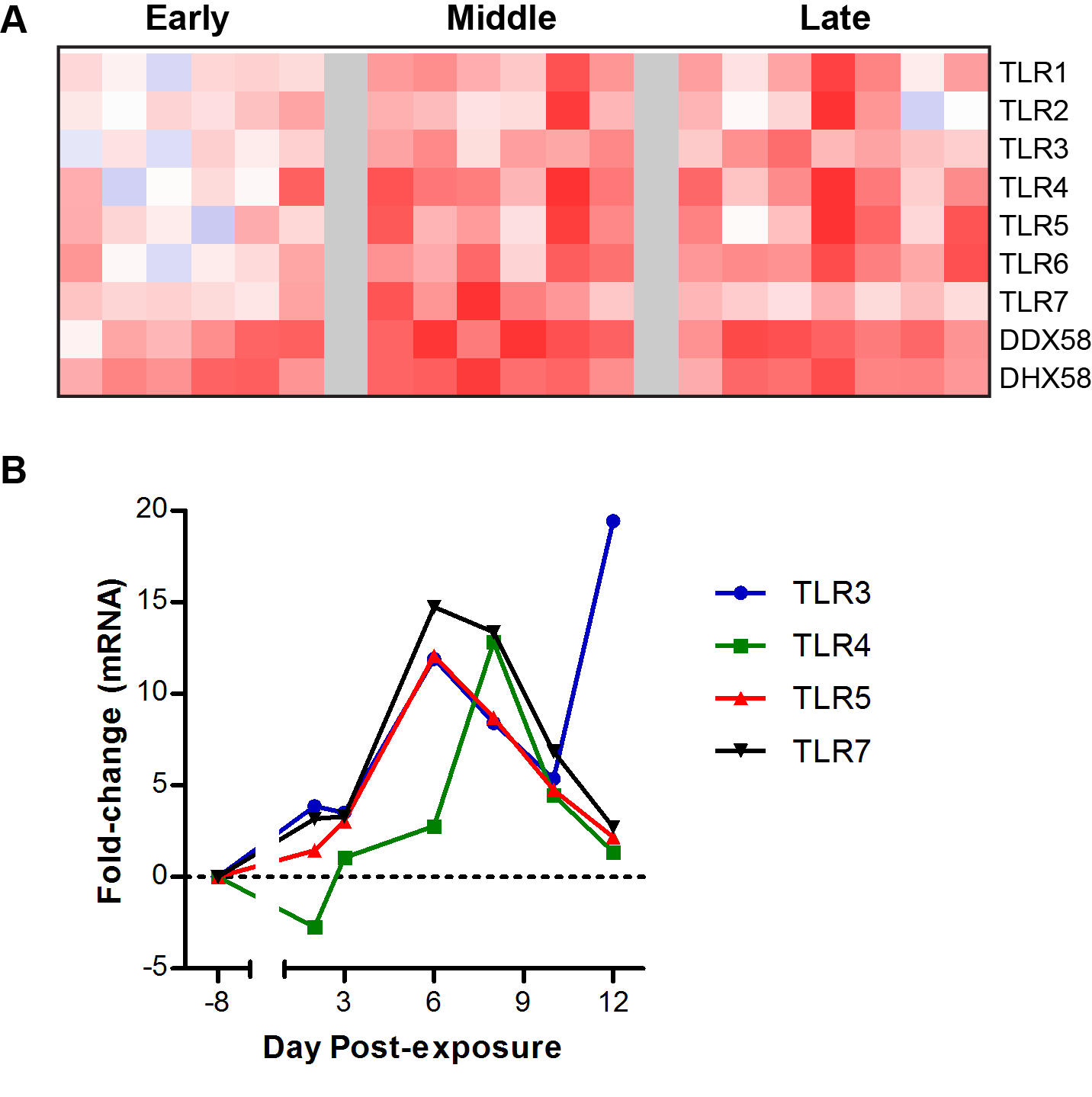

Supplement: Figure S5 — Expression of Toll-like receptors (TLRs) and RIGI-like receptors (RLRs) in LASV-exposed NHPs. (A) Heatmap of TLR1 through 7, DDX58, and DHX58 in the LASV-exposed macaques (DNA microarray). (B) Line graph showing the expression of TLR3 (blue line), TLR4 (green line), TLR5 (red line), and TLR7 (black line) in the PBMCs of LASV-exposed macaques by RT-PCR. (TIF) [file pntd.0002171.s005.tif]

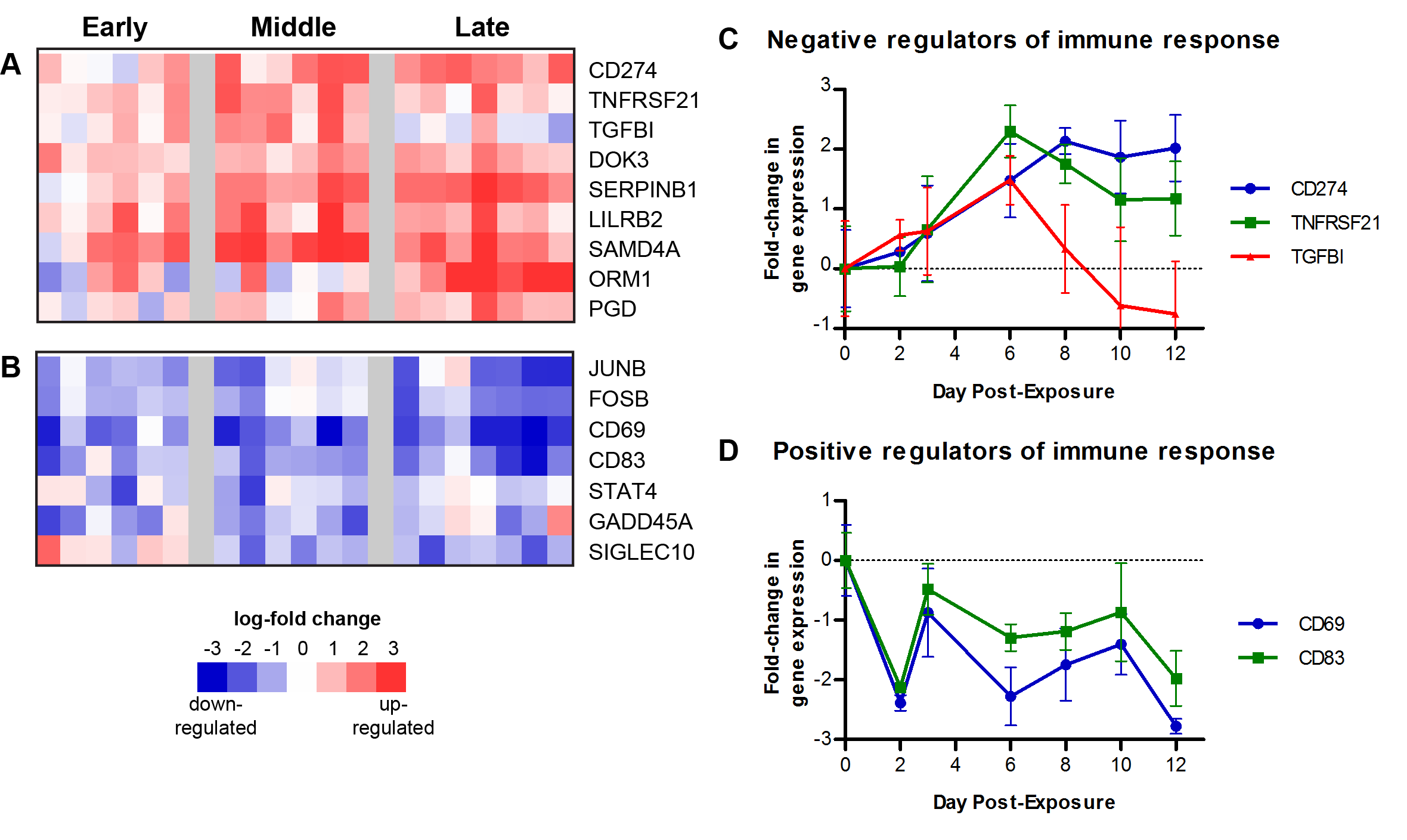

Supplement: Figure S6 — Immunological response. Heatmap of (A) negative and (B) positive regulators of immune response. Line graphs of representative genes from (C) negative regulators represented in A and (D) positive regulators of immune response represented in B, following LASV exposure. (TIF) [file pntd.0002171.s006.tif]
